# Ethical approval processes for research during public health emergencies in Africa: Experiences from a multicountry mpox study

**DOI:** 10.4102/jphia.v17i1.1883

**Published:** 2026-06-25

**Authors:** Mosoka P. Fallah, Olayinka S. Ilesanmi, Nebiyu Dereje, Oluwatoyosi Olawande, Elvis TemFack, Tamrat Shaweno, Abigael A. Mesfin, Cathy A. Tabaro, Shahd O.S. Osman, Yap Boum, Raji Tajudeen, Ngashi Ngongo, Jean Kaseya

**Affiliations:** 1 Directorate of Science and Innovation, Africa Centres for Disease Control and Prevention, Addis Ababa, Ethiopia; 2 Regional Coordination Center, Africa Centres for Disease Control and Prevention, Addis Ababa, Ethiopia; 3 Executive Office, Africa Centres for Disease Control and Prevention, Addis Ababa, Ethiopia

**Keywords:** Africa, approval, ethics, public health, emergencies

## Abstract

Timely initiation of outbreak research is essential for timely public health action. Yet conventional ethics review timelines, often measured in weeks, can delay study start-up and blunt the impact of interventions. During epidemics, routine review timelines of 2 weeks – 9 weeks delay research and weaken public health responses. Fast-tracking ethical review allows researchers to initiate studies at the height of an outbreak, when data are most relevant and informative. A spike in mpox cases across 20 Member States in 2024 prompted the Africa Centres for Disease Control and Prevention (Africa CDC) to declare its first Public Health Emergency of Continental Security. To expedite therapeutic trials, develop supporting policies, and mobilise resources for research and response, the Africa CDC and the World Health Organization (WHO) developed the Research and Innovation pillar within the Continental Incident Management Support Team (IMST). Among key research initiatives was a multicountry social and behavioural study led by Africa CDC. This article includes a synthesis based on implementation experiences from a multicountry mpox socio-behavioural study coordinated by the Africa CDC across 10 African countries. Within a month of IMST’s inauguration, a master protocol and 10 country-specific protocols were finalised, led by Africa CDC in collaboration with national public health institutes and academic experts. Approval processes took 3 weeks – 11 weeks, with most countries completing review within 4 weeks – 8 weeks. This underscores the need to further streamline the ethical review process during emergencies and to improve efficiency.

**Contribution:**

As the global research landscape continues to evolve, there is an urgent need for a unified continental ethical oversight framework to address the historical delays caused by multiple and overlapping requirements.

## Introduction

Timely public health action depends on the prompt beginning of high-quality epidemic research. However, traditional ethics review schedules, which are often measured in weeks, might delay the initiation of a study and diminish the impact of an intervention. The recent Uganda Ebola Sudan outbreak exemplifies how accelerated review is feasible. The World Health Organization (WHO) reported that Uganda’s vaccine trial began ‘with record speed, only 3 days since the outbreak was declared, while ensuring full compliance with international and national regulatory and ethical requirements’.^1^ Likewise, Rwanda leveraged intensive preparedness to launch a Marburg vaccine trial just 10 days after its outbreak began.^2^ Without such speed, research may fail to deliver useful evidence before an epidemic wanes, undermining potential benefits to at-risk populations.

Accelerated ethical review during outbreaks is critical for timely research when data are most relevant. Fast-tracking approvals enable rapid development of diagnostics, treatments, and vaccines, and supports real-time epidemiological studies that inform policy and containment strategies. It minimises bureaucratic delays, prevents duplication, and ensures evidence-based resource allocation, particularly in low-resource settings. Harmonised ethical standards also foster international collaboration. While essential, expedited review must balance speed with scientific, ethical, regulatory, and operational rigour.^3,4,5^ This brief report includes synthesis based on implementation experiences related to obtaining ethical approvals from a multicountry mpox sociobehavioural study coordinated by the Africa Centres for Disease Control and Prevention (Africa CDC) across 10 African countries.^3^

### Background

A key enabler of rapid response has been proactive preparedness. In Uganda’s case, thousands of vaccine doses and trial protocols were pre-positioned in advance of the outbreak, allowing immediate trial activation.^1^ Similarly, Rwandan authorities capitalised on recent simulation exercises and existing partnerships. Having investigational Marburg vaccine doses on hand, they commenced an emergency trial 10 days into the outbreak.^2^ Sierra Leone, confronting mpox, built on reforms from its Ebola experience by establishing special emergency ethics review subcommittees and investing in digital reporting systems. For instance, tools piloted during Ebola now support real-time case reporting and laboratory coordination in Sierra Leone.^4^ Such measures, including pre-approved ‘master’ or adaptive protocols and outbreak-specific standard operating procedures (SOPs) mean that once a pathogen emerges, the contextual protocol can be quickly finalised and submitted for review. Where committees are well-trained and connected to emergency operations, review can proceed swiftly without sacrificing rigour.

In Uganda, Rwanda, and Sierra Leone, expedited procedures and pre-existing infrastructure allowed for quick ethics clearance during outbreaks.^1,3^ Shortly after the 2022 outbreak was declared, Uganda approved a clinical trial for an Ebola Sudan virus vaccine.^1^ Because of pre-positioned doses and quick regulatory procedures, vaccinations arrived in a record 79 days, and trials began in 3 days, and Coordination with WHO for accelerated reviews was made possible by organisations such as the Medical Research Council (MRC) Uganda and Uganda Virus Research Institute, which supplied skilled staff and SOPs.^5,6,7^

During its 2024 outbreak, Rwanda initiated exploratory Marburg vaccine trials, and the ethics committee quickly approved Phase 2 procedures.^3^ The National Ethics Committee treated ethics as an essential response component rather than a postponement and incorporated it into emergency preparation, and timelines were shortened to days with ministerial support and streamlined submissions, in accordance with larger preparedness initiatives.^8,9,10,11^

## Research methods and design

In 2024, Africa CDC declared its first Public Health Emergency of Continental Security (PHECS) in response to a surge in mpox cases across 20 Member States, highlighting the urgent need for coordinated and intensified response efforts.^9^ Africa CDC and WHO established the Research and Innovation pillar under the Continental Incident Management Support Team (IMST) to streamline clinical trials, develop supportive policies, and mobilise resources for research and response. Among key research initiatives was a multicountry social and behavioural study titled ‘Understanding the socio-ecological and behavioural drivers of mpox disease in Africa’, launched in 10 countries (Burundi, Central African Republic [CAR], Congo, Democratic Republic of the Congo [DRC], Kenya, Nigeria, Rwanda, Uganda, Sierra Leone and Côte d’Ivoire) with a target of over 23 000 respondents using a mixed-methods design as part of the first phase of implementation. These countries were in Category 1 and Category 2 priority groups and, therefore, were selected for the initial batch of countries for rapid study rollout. The study period covered from November 2024 to December 2025. Data on ethical approval timelines were compiled from study coordination records, including documented submission and approval dates from national ethics committees and institutional review boards. These data were cross-verified with country teams to ensure accuracy and consistency prior to analysis and presentation in Table 1.

**TABLE 1:** Ethical approval submission date and approval processes timeline in nine countries involved in the Africa centres for disease control and prevention mpox socio-behavioural study in Africa.

№	Country	Research Ethical Committee (REC) authority & process	Payment required	Submission date	Second submission date	Approval date	Duration from submission to approval (days)	Remarks
1	DRC	Institute of Public Health (INSP); ethical approval followed by INSP registration	Yes	15 September 2024	Not applicable	25 October 2024	40	Approval and registration required payment
2	Congo	National ethics authority	Yes	24 October 2024	Not applicable	14 November 2024	21	Standard ethical review process
3	Kenya	National ethics committee	Yes	04 December 2024	Not applicable	30 January 2025	57	Separate research permit required
4	Rwanda	Rwanda National Ethics Committee (RNEC)	Yes	27 November 2024	25 January 2025	11 February 2025	76	Initial feedback within 2 weeks; presentation held 07 December 2024
5	Burundi	National ethics committee	Yes	19 January 2025	Not applicable	14 February 2025	26	Study could not commence until statistical visa obtained
6	Uganda	MUREC → Uganda Council of Science and Technology (UCST)	Yes	09 January 2025	17 February 2025	25 February 2025	47	UCST provides final approval; PI creates online account
7	Nigeria	National Health Research Ethics Committee (NHREC)	No	27 January 2025	Not applicable	28 February 2025	32	Initial email unacknowledged; expedited approval later confirmed
8	Côte d’Ivoire	National ethics committee	Yes	28 February 2025	Not applicable	04 April 2025	35	Standard review process
9	Central African Republic (CAR)	National ethics committee	Yes	18 February 2025	Not applicable	14 April 2025	55	Verbal consent allowed training of field team before approval
10	Sierra Leone	National ethics committee	Yes	09 June 2025	Not applicable	03 July 2025	24	Review on expedited basis

PI, Principal investigator; MUREC, Makerere University Research Ethics Committee; DRC, Democratic Republic of the Congo; CDC, Centres for Disease Control and Prevention.

### Ethical considerations

This article followed all ethical standards for research without direct contact with human or animal subjects.

## Results

Within a month of IMST’s inauguration, a master protocol and 10 country-specific protocols were finalised, led by Africa CDC’s Directorate of Science and Innovation in collaboration with national public health institutes and academic experts. The approval processes took from 3 weeks – 11 weeks, with most countries completing review within 4 weeks – 8 weeks. The experiences of the countries involved are summarised in Table 1.

Several countries required multiple layers of approval, further extending timelines. For example, in Uganda, approval involved both Makerere University Research Ethics Committee (MUREC) and the Uganda Council of Science and Technology (UCST), while in the DRC, ethical approval had to be followed by registration with the Institute of Public Health. Rwanda required a second submission and an in-person presentation, although it demonstrated relatively strong procedural efficiency by providing initial feedback within 2 weeks. At the time of writing, most participating countries have successfully completed data collection for the multicountry mpox socio-behavioural study coordinated by the Africa CDC. Despite variations in ethical approval timelines and procedural requirements across countries, all study activities were conducted in full compliance with national and institutional ethical standards. Importantly, there were no reported ethical compromises throughout the implementation of the study. Quick adaptation to the outbreak scenario while maintaining ethical standards was made possible by pre-approved adaptive protocols.

## Discussion

The absence of harmonised standards, clear expedited pathways, and coordinated multicountry review mechanisms contributes to delays that could undermine timely evidence generation during outbreaks. This underscores the need for further streamlining of the ethical review process during emergencies and for efficiency improvements.

The experience of non-uniform submission and delay in receiving ethical approval during public health emergencies poses unique ethical dilemmas that require urgent attention and a robust ethical review framework that minimises delays while ethical standards are not compromised. Ethical frameworks must be adaptive to address emergent issues effectively, yet the current literature reveals a significant gap in understanding the ethics review processes specific to African contexts during public health emergencies. Obtaining ethical approval for multicountry studies during emergencies in Africa remains a major challenge.

Several challenges have been raised regarding delayed approval processes. High workloads and inadequate compensation discourage member participation, ultimately affecting the quality of ethical reviews. Many research ethical committee (REC) members report difficulties, including insufficient administrative support, limited knowledge of research ethics, and a lack of resources to adequately review complex research proposals, particularly those involving coronavirus disease 2019 (COVID-19) studies.^10^ On the other hand, in the multicountry mpox study, countries that demonstrated better performance in ethical approval timelines were those with streamlined, single-layered review processes and fewer administrative requirements. These examples show that, with advance planning, review and approval can occur in days rather than the 2 weeks – 9 weeks typical of routine processes.^11^

### Limitations

This brief report focuses on a single multicountry mpox research initiative coordinated by the Africa CDC, which can be a limitation. While this provides valuable real-world insights, the findings may not be fully generalisable to other disease contexts, regions, or types of studies, particularly those with different regulatory or operational complexities. Descriptive synthesis was used in this report with no comparative or statistical methods. Therefore, no inferential conclusions can be drawn from the findings.

### Recommendations and future directions

Development of national SOPs for emergency ethics reviews has been recommended as a way forward to address these issues.^12^ Pre-review generic protocols and creation of model templates for rapid customisation have been suggested.^5^ In addition, the establishment of regional platforms such as African Vaccines Regulatory Forum (AVAREF) extensions for multicountry harmonised reviews could also alleviate the challenges.^12^ Promoting data and sample sharing agreements and clear benefit plans pre-outbreak would harmonise stakeholders. Furthermore, training REC members on outbreak ethics, including digital tools and stakeholder coordination, could support rapid ethical processes.^12^ Integrating ethics into national health emergency plans with political support could prevent delays in approval.^13,14^

The establishment of a Continental Health Research Ethics Committee (CH-REC) in Africa has emerged as a pivotal response to the unique ethical challenges faced by the continent in research practices. An Africa-centric framework for ethical research practices that ensures optimal protection of participants in complex and diverse cultural and socioeconomic settings of Africa has been proposed.^15^ The CH-REC aims to enhance ethical standards, promote fairness in research collaborations, and ensure that local voices are represented in global dialogues is thereby fostering trust and integrity within research communities, while the sovereignty of member states is maintained.

## Conclusion

Getting ethical approval for multicountry studies during public health emergencies in Africa has been a major challenge. As the global research landscape continues to evolve, particularly in the wake of public health emergencies such as the COVID-19 pandemic and the mpox pandemic. Experiences from Africa CDC’s multicountry mpox research can be used as a reference when conducting research during outbreaks, and it underscores the need for further streamlining of the ethical review process during emergencies. As Africa strives to enhance its research capabilities and collaborate with international partners, the establishment of a CH-REC represents a significant step towards ensuring ethical integrity and promoting equitable research practices that reflect the continent’s unique values and needs.
